# A maximum pseudo-likelihood approach for phylogenetic networks

**DOI:** 10.1186/1471-2164-16-S10-S10

**Published:** 2015-10-02

**Authors:** Yun Yu, Luay Nakhleh

**Affiliations:** 1 Department of Computer Science, Rice University, Houston, 77005 Texas, USA; 2 Department of BioSciences, Rice University, Houston, 77005 Texas, USA

## Abstract

**Background:**

Several phylogenomic analyses have recently demonstrated the need to account simultaneously for incomplete lineage sorting (ILS) and hybridization when inferring a species phylogeny. A maximum likelihood approach was introduced recently for inferring species phylogenies in the presence of both processes, and showed very good results. However, computing the likelihood of a model in this case is computationally infeasible except for very small data sets.

**Results:**

Inspired by recent work on the pseudo-likelihood of species trees based on rooted triples, we introduce the pseudo-likelihood of a phylogenetic network, which, when combined with a search heuristic, provides a statistical method for phylogenetic network inference in the presence of ILS. Unlike trees, networks are not always uniquely encoded by a set of rooted triples. Therefore, even when given sufficient data, the method might converge to a network that is equivalent under rooted triples to the true one, but not the true one itself. The method is computationally efficient and has produced very good results on the data sets we analyzed. The method is implemented in PhyloNet, which is publicly available in open source.

**Conclusions:**

Maximum pseudo-likelihood allows for inferring species phylogenies in the presence of hybridization and ILS, while scaling to much larger data sets than is currently feasible under full maximum likelihood. The nonuniqueness of phylogenetic networks encoded by a system of rooted triples notwithstanding, the proposed method infers the correct network under certain scenarios, and provides candidates for further exploration under other criteria and/or data in other scenarios.

## Background

The last decade has seen an explosion in the development of methods for inferring species trees from genome-wide data in the presence of incomplete lineage sorting (ILS); see [1] for a recent review. Indeed, ILS has been shown to be at play in various phylogenomic data sets; e.g., [2-4]. In the presence of ILS, the species phylogeny still takes the shape of a tree, with the difference gene trees "growing" within its branches. Another evolutionary process that results in gene tree incongruence in eukaryotic data sets, but violates the tree shape of the species phylogeny, is hybridization. Hybridization--the mating of individuals from different species--is believed to play an important role in several groups of eukaryotic species [5-9]. It has been estimated that at least 25% of plant species and 10% of animal species hybridize [7]. The non-treelike phylogenetic relationships resulting from hybridization are best modeled by *phylogenetic networks*.

Recent studies have reported patterns of co-occurrence of hybridization and ILS [10-14]. These studies call for developing methods that account *simultaneously *for ILS and hybridization. In recent years, some efforts have been made to address this issue, but they all focused on limited special cases of phylogenetic networks [15-20]. More recently, methods have been developed for general phylogenetic networks, including maximum parsimony [21], maximum likelihood [22-24] and distance-based methods [25]. Of these, maximum likelihood produces the most accurate results and allows for estimating, in addition to the network topology, branch lengths and other parameters.

Computing the likelihood of a phylogenetic network under the models of [22,24] is computationally very expensive. When this step is coupled with a search heuristic that traverses the space of phylogenetic networks and other parameters, application of maximum likelihood becomes limited to very small data sets (fewer than 10 taxa and 3 reticulations). In this paper, we propose a maximum pseudo-likelihood approach for inferring phylogenetic networks in the presence of hybridizations and ILS. The work extends MP-EST, which is a maximum pseudo-likelihood approach for estimating species trees from a collection of gene trees under the multispecies coalescent model [26]. The pseudo-likelihood of a species tree is computed based on the frequencies of rooted triples in the input gene trees. Given that a tree is uniquely encoded by its set of rooted triples, the method of [26] has theoretical guarantees of convergence, in addition to its empirical performance. However, a phylogenetic network is not necessarily uniquely encoded by its triple set. The implication of this fact is that our method might not identify the true network (even when given sufficiently large amounts of data), but one that is equivalent to it in terms of the rooted triples it induces. However, it is important to note that a phylogenetic network could very well be uniquely encoded by a system of rooted triples. Further, when the phylogenetic network is not uniquely encoded by a system of rooted triples, the networks that the method infers could be explored using other criteria (e.g., under likelihood based on gene trees) and/or other types of data (e.g., gene trees with branch lengths and molecular sequences).

We have implemented the method in the open-source software package PhyloNet [27], which can be accessed at [28]. We analyzed the performance of the method on a biological data set as well as simulated data. Results on these data sets show that the method has a very good performance in terms of accuracy of the inferred evolutionary histories, as well as computational requirements. This method will enable analyses of larger data sets than is currently feasible where hybridization and ILS are suspected to be at play.

## Methods

Liu *et al*. recently introduced MP-EST, a maximum pseudo-likelihood approach for estimating species trees from a collection of rooted gene trees under the multispecies coalescent [26]. The method resulted in significant improvements in the running time of statistical inference of species trees. Inspired by this work, we propose a method for estimating species phylogenies in the presence of both hybridization and incomplete lineage sorting under maximum pseudo-likelihood.

### Phylogenetic networks, gene trees, and rooted triples

A (binary) phylogenetic network [29] Ψ on set  X of species (taxa) is a rooted, directed, acyclic graph whose node-set is *V *(Ψ) = {*r*} *∪ V_L _∪ V_T _∪ V_N _*where

• *r *is the root of Ψ and satisfies *d^−^*(*r*) = 0 and *d*^+^(*r*) = 2;

• *V_L_*: the leaf-set bijectively labeled by  X, where *d^−^*(*v*) = 1 and *d*^+^(*v*) = 0 for any *v ∈ V_L_*;

• *V_T _*: internal tree nodes, where *d^−^*(*v*) = 1 and *d*^+^(*v*) = 2 for any *v ∈ VT *; and,

• *V_N _*: reticulation nodes, where *d^−^*(*v*) = 2 and *d*^+^(*v*) = 1 for any *v ∈ V_N._*

Here, *d^−^*(*v*) and *d*^+^(*v*) are the in-degree and out-degree of node *v*, respectively. We denote by *E*(Ψ) the set of edges in network Ψ. The phylogenetic network has branch lengths *λ *: *E*(Ψ) *→ *ℝ^+^. Hereafter, we will use Ψ to denote both the topology and branch lengths of a phylogenetic network. Further, as in [22,24], for a probabilistic setting, there is an additional function, referred to as the inheritance probability, *γ *: *E*(Ψ) *→ *[0, 1] that satisfies:

• *γ*(*e*) = 1 for every edge *e *whose head is a tree node, and

• *γ*(*e*1) + *γ*(*e*2) = 1 for every pair of edges *e*1 and *e*2 whose head is the same reticulation node.

In [24], we discussed how to generalize the function *γ *so that it varies across loci, and that generalization would be trivial to incorporate in the methods below.

**Figure 1:**
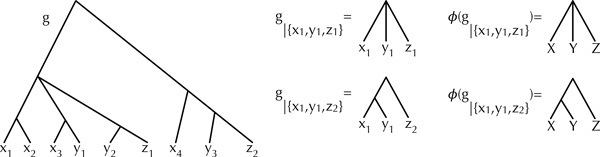
**Gene trees and rooted triples**. A gene tree *g *on three species *X, Y *, and *Z*, where multiple alleles are sampled per species. Two induced triples by the gene tree and their mapping to the species names are shown.

A gene tree *g *on set  X of species is a rooted tree (not necessarily binary) whose leaves are labeled (not necessarily bijectively) by  X. To distinguish the leaves that are labeled by the same element of  X, we add subscripts to the leaf labels. Figure 1 shows a gene tree *g *on set *X *= {*X*, *Y*, *Z*} of species, where four alleles are sampled from species *X *(labeled *x*_1_*, ..., x*_4_), three alleles are sampled from species *Y *(labeled *y*_1_*, ..., y*_3_), and two alleles are sampled from species *Z *(labeled *z*_1 _and *z*_2_). In particular, in this work we allow a gene tree to have zero alleles sampled from some species.

A rooted triple (from now on we will just write "triple", since we only deal with rooted topologies) is a rooted tree with three leaves. If the triple is binary, we write *xy|z *to denote that the triple puts *x *and *y *closer to each other than either of them to *z*. If the triple is nonbinary, then it is *xyz*. We denote by *g|*{*x,y,z*} the triple in the gene tree *g *induced by restricting its leaf-set to the three leaves labeled *x, y*, and *z*. Figure 1 shows the two triples induced by {*x*_1_*, y*_1_*, z*_1_} and {*x*_1_*, y*_1_*, z*_2_}. Finally, to link the leaf-labels in the gene tree to their corresponding taxa in the phylogenetic network, we introduce function *φ *which maps an allele label in the gene tree to its corresponding taxon in the network. For example, in Figure 1, *φ*(*z*_1_) = *φ*(*z*_2_) = *Z*. Further, we use *φ*(*g|*{*x,y,z*}) to denote the induced triple with its leaf-labels replaced by the taxa names (of species). Figure 1 illustrates *φ*.

### Pseudo-likelihood of a species network

Let  X be a set of taxa (species), *t *= *XY |Z *be a *binary *triple with *X, Y, Z ∈* X, and *g *be a gene tree on  X. We denote by *a*(*g, X*), for *X *∈ *X*, the set of alleles from *X *that label leaves of *g*. For example, in Figure 1, *a*(*g, X*) = {*x*_1_*, x*_2_*, x*_3_*, x*_4_}. We define *ρ*(*t, g*) to be the number of times *t *is induced by *g *(when the leaf-labels are mapped to  X using the function *φ*) normalized by the number of times any triple on *X, Y *, and *Z *is induced by *g*. Clearly, if at most one allele per species is sampled in *g*, then any triple is either not induced by the gene tree or induced once. However, since we allow multiple alleles per species, this might not be the case. Note that while *t *is binary, it could be the case that *g|*{*x_i _,y_j _,z_k_*} is nonbinary. Since there are three ways of resolving a nonbinary triple, a nonbinary triple *g|*{*x_i _,y_j _,z_k_*} contributes 1*/*3 to the value of *ρ*(*t, g*). Accounting for these two issues, *ρ*(*t, g*) for *t *= *XY |Z *equals

(1) $$\frac{\underset{x\in a\left(g,X\right),y\in a\left(g,Y\right),z\in a\left(g,Z\right)}{\sum }\left(I\text{(}\phi \text{(}g\text{|}{}_{\left\{x,y,z\right\}}\text{) = }XY|Z\right)\cdot 1+\text{I}(\phi \text{(}g|{}_{\left\{x,y,z\right\}})=XYZ)\cdot 1/3)}{\left|a\left(g,X\right)|\cdot |a\left(g,Y\right)|\cdot |a\left(g,Z\right)\right|},$$

where  I is the indicator function defined by $I\left(e\right)=\text{1}$ when *e *is true and $I\left(e\right)=\text{0}$ when *e *is false. For a set *G *of gene trees, we define $\rho \left(t,G\right)=\sum_{g\in G}\rho \left(t,g\right)$. If the denominator in Eq. (1) equals zero, we set *ρ*(*t, g*) = 0.

Given a set *G *of gene trees, the three binary triples *t*_1 _= *XY|Z, t*_2 _= *XZ|Y*, and *t*_3 _= *YZ|X *on a set {X,Y,Z}⊆X have a multinomial distribution given by

(2) $$f\left(\rho \left(t_{1},G\right),\rho \left(t_{2},G\right),\rho \left(t_{3},G\right)|\Psi ,\gamma \right)=\frac{|G|!}{\overset{3}{\underset{i=1}{\prod }}\rho \left(t_{i},G\right)!}\overset{3}{\underset{i=1}{\prod }}{\left(P\left(t_{i}|\Psi ,\gamma \right)\right)}^{\rho \left(t_{i},G\right)},$$

where *P *(*t|*Ψ*, γ*) is the probability of rooted triple *t *given network Ψ and inheritance probabilities *γ *[22,21].

Finally, the pseudo-likelihood of phylogenetic network Ψ and inheritance probabilities *γ *given a set *G *of gene trees is given by

$$L\left(\Psi ,\gamma |G\right)=\underset{\left\{X,Y,Z\right\}\subseteq X}{\prod }f\left(\rho \left(XY|Z,G\right),\rho \left(XZ|Y,G\right),\rho \left(YZ|X,G\right)|\Psi ,\gamma \right).$$

A maximum pseudo-likelihood approach seeks Ψ*^∗ ^*and *γ^∗ ^*that maximize Eq. (3).

Since for a given set *G *of gene trees $\frac{|G|!}{\prod_{i=1}^{3}\rho \left(t_{i},G\right)!}$ is a constant irrespective of Ψ and *γ*, this term is dropped from the pseudo-likelihood computation when searching for Ψ*^∗ ^*and *γ^∗^*.

### Convergence and identifiability

It follows from the strong law of large numbers [30] that as the number of gene trees

*|G| *goes to infinity, the proportions of rooted triples in gene trees converge to their

expectations, that is

(4) $$\left\{\frac{\rho \left(t_{1},G\right)}{\left|G\right|},\frac{\rho \left(t_{2},G\right)}{\left|G\right|},\frac{\rho \left(t_{3},G\right)}{\left|G\right|}\right\}\overset{a.s.}{\underset{}{\to }}\left\{P\left(t_{1}|\hat{\Psi },\hat{\gamma }\right),P\left(t_{2}|\hat{\Psi },\hat{\gamma }\right),P\left(t_{3}|\hat{\Psi },\hat{\gamma }\right)\right\},$$

where $\hat{\Psi }$ is the true phylogenetic network and $\hat{\gamma }$ are the true inheritance probabilities. Thus, as *|G| *goes to infinity, *L*(Ψ*, γ|G*) converges to

(5) $$H\left(\Psi ,\gamma \right)=\underset{\left\{X,Y,Z\right\}\subseteq x}{\prod }\left(\frac{|G|!}{\prod_{i=1}^{3}(|G|\cdot P\left(t_{i}|\hat{\Psi },\hat{\gamma }\right)!}\overset{3}{\underset{i=1}{\prod }}{\left(P\left(t_{i}|\Psi ,\gamma \right)\right)}^{|G|\cdot P(t_{i}|\hat{\Psi },\hat{\gamma })}\right)$$

A phylogenetic tree is uniquely encoded by its triple system [31]. More specifically, given a phylogenetic tree *T *, let *R*(*T*) be the set of triples induced by tree *T *. Then no tree *T′ *exists such that *T ≠ T′ *and *R*(*T*) = *R*(*T′*). Combining this fact with Eq. (5) and the fact that *H*(Ψ*, γ*) is maximized when $\lambda =\hat{\lambda }$ and $\gamma =\hat{\gamma }$, it is clear that when the species phylogeny Ψ is a tree, as *|G| *goes to infinity, Ψ*^∗ ^*converges to the true species tree [26].

**Figure 2:**
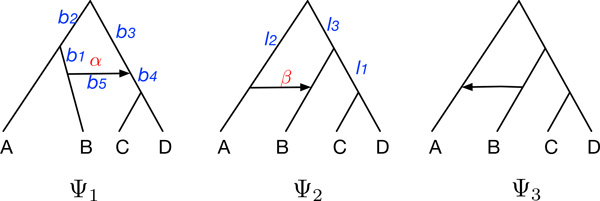
**Illustration of the lack of network identifiability under the proposed pseudo-likelihood framework**. Three phylogenetic networks with the same set of triples: *A|BC, AB|C, A|BD, AB|D, A|CD*, and *B|CD*. Branch lengths and inheritance probabilities are shown in blue and red, respectively, for Ψ_1 _and Ψ_2_.

However, in contrast to trees, triples do not necessarily uniquely encode a phylogenetic network [32]. For example, the three phylogenetic networks Ψ_1_, Ψ_2 _and Ψ_3 _in Figure 2 have different topologies, but they induce (a network induces a triple if at least one of the trees displayed by the network induces that triple) the same triple system {*A|BC, AB|C, A|BD, AB|D, A|CD, B|CD*}. This means that, given a phylogenetic network Ψ (topology and branch lengths) and inheritance probabilities *γ*, if there is a phylogenetic network Ψ′ s.t. *R*(Ψ) = *R*(Ψ′) (which is not necessarily always true), then there exist branch lengths for Ψ′ and inheritance probabilities *γ*′ such that *P *(*t|*Ψ*, γ*) = *P *(*t|*Ψ′*, γ*′) for every rooted triple *t*. For example, in Figure 2, given network Ψ1 with its branch lengths and inheritance probabilities, we can obtain *P *(*t|*Ψ_1_*, γ*_1_) = *P *(*t|*Ψ_2_*, γ*_2_) for every triple *t *by setting the branch lengths of network Ψ_2 _and inheritance probabilities as

$$\begin{array}{c} l_{1}=-\text{ln}\left(\alpha e^{b_{2}}+\left(1-\alpha \right)e^{b_{5}}\right)+b_{5}+b_{4}+b_{2},\\ l_{{}_{2}}=-\text{ln}((\alpha e^{b_{2}+b_{3}}\left(e^{b_{1}+b_{5}}\left(3\alpha e^{b_{2}}+3-4\alpha \right)-e^{b_{1}}-\alpha e^{b_{2}}-e^{b_{5}}+\alpha \right)+\\ \left(1-\alpha \right)e^{b_{1}+b_{5}}\left(e^{b_{2}}\left(1-\alpha +\alpha e^{b_{2}}\right)-\left(1+\alpha \right)e^{b_{3}}\right))/\\ \left(\alpha e^{b_{1}+b_{2}+b_{3}}\left(3e^{b_{5}}-1\right)+e^{b_{1}+b_{5}}\left(\left(1-\alpha \right)e^{b_{2}}-e^{b_{3}}\right)-\alpha e^{b_{3}+b_{5}}\right)))+b_{2},\\ l_{3}=\text{ln}\left(\frac{\left(\alpha -1\right)e^{b_{5}}-\alpha e^{b_{2}}}{\alpha e^{b_{1}+b_{5}}\left(e^{b_{2}}\left(3\left(\alpha -1\right)e^{b_{3}}-\alpha \right)+\left(1-\alpha \right)e^{b_{3}}\right)-{\left(1-\alpha \right)}^{2}e^{b_{2}+b_{3}}}\right)+b_{1}+b_{3}, \end{array}$$

and

$$\beta =\frac{\left(1-\alpha \right)(\alpha e^{b_{1}+b_{2}+b_{3}}\left(-3e^{b_{5}}+1\right)-e^{b_{1}+b_{5}}\left(\left(1-\alpha \right)e^{b_{2}}-e^{b_{3}}\right)+\alpha e^{b_{3}+b_{5}}}{\left(1-\alpha \right)e^{b_{1}+b_{3}+b_{5}}\left(-3\alpha e^{b_{2}}+1+\alpha \right)-e^{b_{1}+b_{2}}\left({\left(1-\alpha \right)}^{2}e^{b_{5}}-\alpha e^{b_{3}}\right)-\alpha^{2}e^{b_{2}+b_{3}}}.$$

A concrete example of these settings is:

• network Ψ_1_: *b*_1 _= 1, *b*_2 _= 1, *b*_3 _= 2, *b*_4 _= 1, *b*_5 _= 0, *α *= 0.1

• network Ψ_2_: *l*_1 _= 1.841435, *l*_2 _= 1.951019, *l*_3 _= 0.207841, *β *= 0.6631633.

This result means that when a species network Ψ is not uniquely encoded by its triple system, as the number of gene trees *|G| *goes to infinity, argmax_Ψ,γ _*L*(Ψ*, γ|G*) is not unique, and one of the solutions is the true species network $\hat{\Psi }$ and true inheritance probabilities $\hat{\gamma }$. This leads to an issue in our inference: if the optimal phylogenetic network $\hat{\Psi }$ is not uniquely encoded by its triple system $R\left(\hat{\Psi }\right)$, the maximum pseudo-likelihood search might return any of the optimal networks with the same triple system. To ameliorate (yet, not guaranteed to always solve) the identifiability issue, one heuristic is to save all optimal networks identified during the search based on pseudo-likelihood and then optimize their branch lengths and inheritance probabilities using the full likelihood computation [22,21] to identify the optimal one among them. However, it is important to keep in mind that full likelihood computation can be infeasible except for very small data sets.

### Searching for Ψ*^∗ ^*and *γ^∗^*

Given a set of gene trees *G*, Ψ*^∗ ^*and *γ^∗ ^*that maximize *L*(Ψ*, γ|G*) are searched by traversing the space of phylogenetic networks and inheritance probabilities using simulated annealing. The search starts from initial values of Ψ and *γ *and in every iteration, one of the following operations is selected randomly according to their preset weights:

• Modifying one or more branch lengths.

• Modifying one or more inheritance probabilities.

• Adding a reticulation edge.

• Deleting a reticulation edge.

• Relocating the head of a reticulation edge.

• Relocating the tail of an edge.

The first two operations do not change the topology of the network. Full details of how these operations are implemented are given in [24]. During the search, if the new network has higher pseudo-likelihood than the current one, it is always accepted; otherwise, it is accepted with some probability. The search terminates if one of two conditions is satisfied: (1) the number of iterations reaches some preset maximum value or (2) the search is alternating between a collection of species networks with high pseudo-likelihoods, and a sufficient number of iterations have passed since visiting any other species networks. The details of how the probability of acceptance is set and the termination conditions are determined are similar to those used in [33,34].

Since branch lengths and inheritance probabilities are sampled, rather than optimized, during the search, some solutions could be missed due to this sampling. One heuristic to ameliorate this problem is to keep the top *k *optimal networks during the search, and then at the end optimize the branch lengths and inheritance probabilities (under the pseudo-likelihood criterion) of only these networks to identify the optimal one. We implemented this in our method and we discuss its performance in the simulation results below.

## Results

### A yeast data set

Using our method, we reanalyzed the yeast dataset of [35]. It contains 1070 genes from 23 yeast genomes. We rooted the gene trees under the MDC criterion using the algorithms of [36,37] and the species tree reported in [35] which was inferred by both maximum-likelihood and Bayesian inference on their concatenated sequence alignment. It is worth mentioning that all 1070 gene trees were topologically distinct and none of them agreed with the inferred species trees.

**Figure 3:**
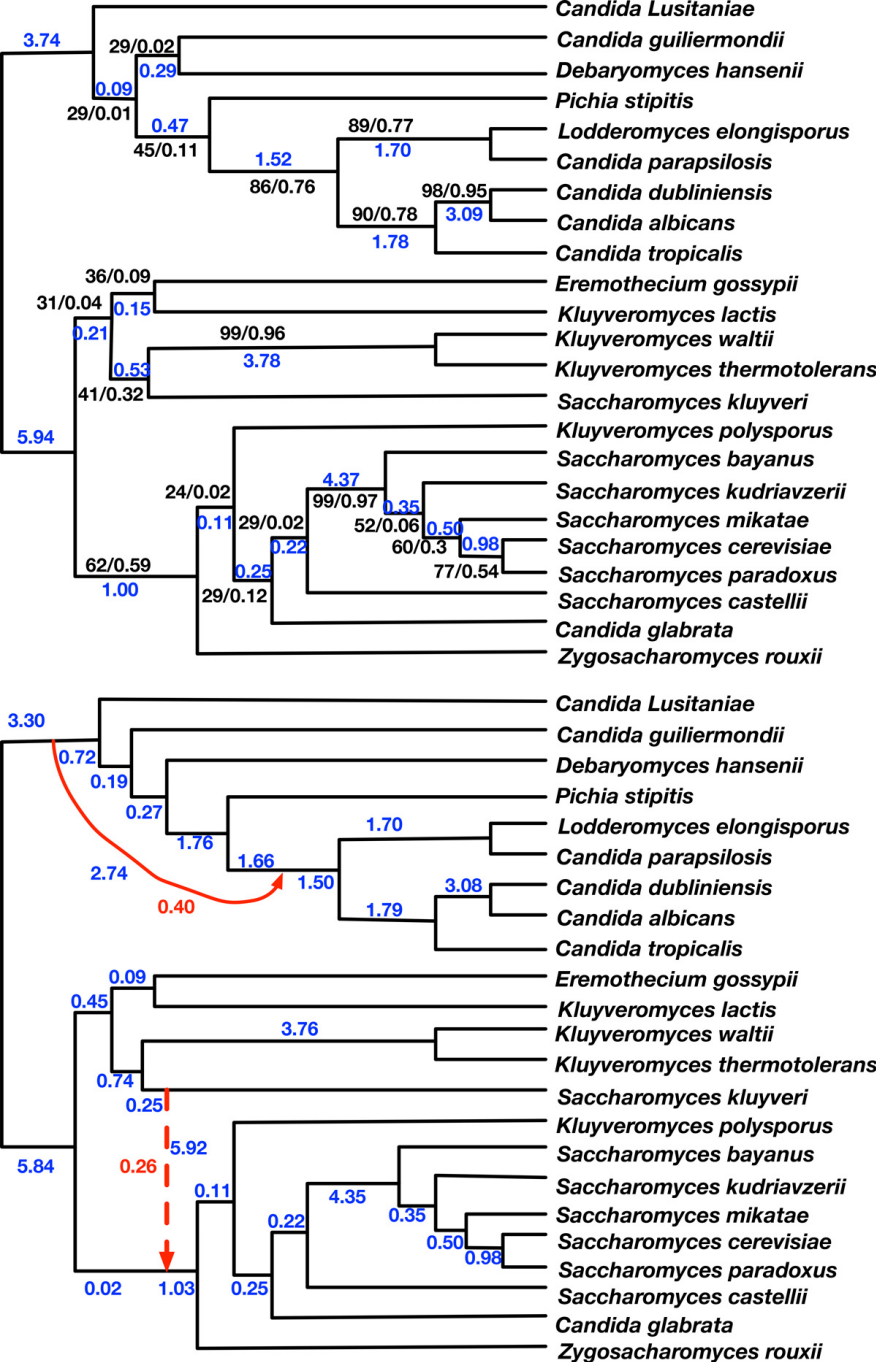
**Reanalysis of the 1070-gene yeast data set of **[35]. Top: the species tree inferred by maximum pseudo-likelihood when no reticulations are allowed during the search. It is identical to the tree reported in [35]. The two black numbers for every internal node are gene-support frequency (left) and internode certainty (right) reported in [35]. Bottom: the species network inferred by maximum pseudo-likelihood with 2 reticulations. The red solid edge is the reticulation edge in the optimal species network with 1 reticulation. Blue and red numbers are branch lengths and inheritance probabilities, respectively, inferred by the method.

The optimal species networks with 0, 1 and 2 reticulations inferred by our method are shown in Figure 3. Their log pseudo-likelihoods are *−*324904, *−*323034, and *−*321710, respectively. The optimal species tree is the same as the one inferred in [35]. The optimal network with 3 reticulations (not shown here) has lower pseudo-likelihood than the one with 2 reticulations, so our method proposes the optimal network with 2 reticulations, shown at the bottom of Figure 3, as the hypothesis for the evolutionary history of this dataset.

In the species tree reported in Figure 3, descending from the MRCA of all the *Candida *species are two successive branches with very poor support (29*/*0.01 and 29*/*0.02). Further, the total time on the path from the MRCA of *C. parapsilosis *and *C. albicans *to the MRCA of all *Candida *species is 1.52 + 0.47 + 0.09 = 2.08 coalescent units. On the other hand, an analysis that accounts for the possibility of hybridization (in addition to incomplete lineage sorting) estimates longer branches, making the same path of length 1.50 + 1.66 + 1.76 + 0.27 + 0.19 + 0.72 = 6.1 coalescent units, which is almost three times as long. Further, it estimates an inheritance probability of 0.4 at the newly added reticulation edge. In other words, this combination of a new reticulation edge and much longer path indicate that much of the incongruence in this part of the tree can be explained by hybridization, rather than incomplete lineage sorting. Notice that inferring this reticulation edge also grouped *D. hansenii *differently, which is one of the two clades with very low support in the species tree. This new reticulation also posits that many of the gene trees indicate that *D. hansenii *and *P. stipitis *are much more recent descendants from the MRCA with *Candida *due to hybridization. Finally, for this part of the network, observe that the reticulation edge has a non-negligible length of 2.74 coalescent units. This implies the possibility that the hybridization involved a sister species of the MRCA of the *Candida *species that was not sampled in this data set.

A similar scenario can be observed in the other part of the species phylogeny (with the *Kluyveromyces *and *Saccharomyces *species). In this part, the underlying "tree" grouping does not differ from that in the estimated species tree, and the branch lengths are also very similar. However, the new inferred reticulation edge (the dashed one) groups *S. kluyveri *with the other clade that has all the other *Saccharomyces*, and this edge is very long (5.92 coalescent units). This indicates that this evolutionary history supports grouping *S. kluyveri *with the other clade, yet with hardly any incomplete lineage sorting involved in this grouping.

In other words, the new analysis, which is enabled by the fast computation of pseudo-likelihood of networks, supports a hypothesis of (at least) two major hybridization events in this data set and more divergence in certain parts of the phylogeny than is supported by the species tree.

### Simulated data

We also used synthetic data to test the performance of our method in terms of accuracy. We used the phylogenetic network in Figure 3 with an added outgroup as the model species phylogeny. Within the branches of this network, we simulated 100, 250, 500, 1000 and 2000 gene trees using the program ms [38]. For each number of gene trees, 30 datasets were generated. Then down each gene tree we used seq-gen [39] to generate sequences of lengths 250, 500 and 1000 under the GTR model of sequence evolution. We set the population mutation rate to 0.036, the base frequencies of the nucleotides A, C, G and T to 0.2112, 0.2888, 0.2896, and 0.2104, respectively, and the relative rates of substitutions to 0.2173, 0.9798, 0.2575, 0.1038, 1 and 0.2070. At last, gene trees were reconstructed using RAxML [40] and rooted at the outgroup. For each sequence alignment, RAxML was run five times and the best tree among these five runs was used as the estimated gene tree.

**Figure 4:**
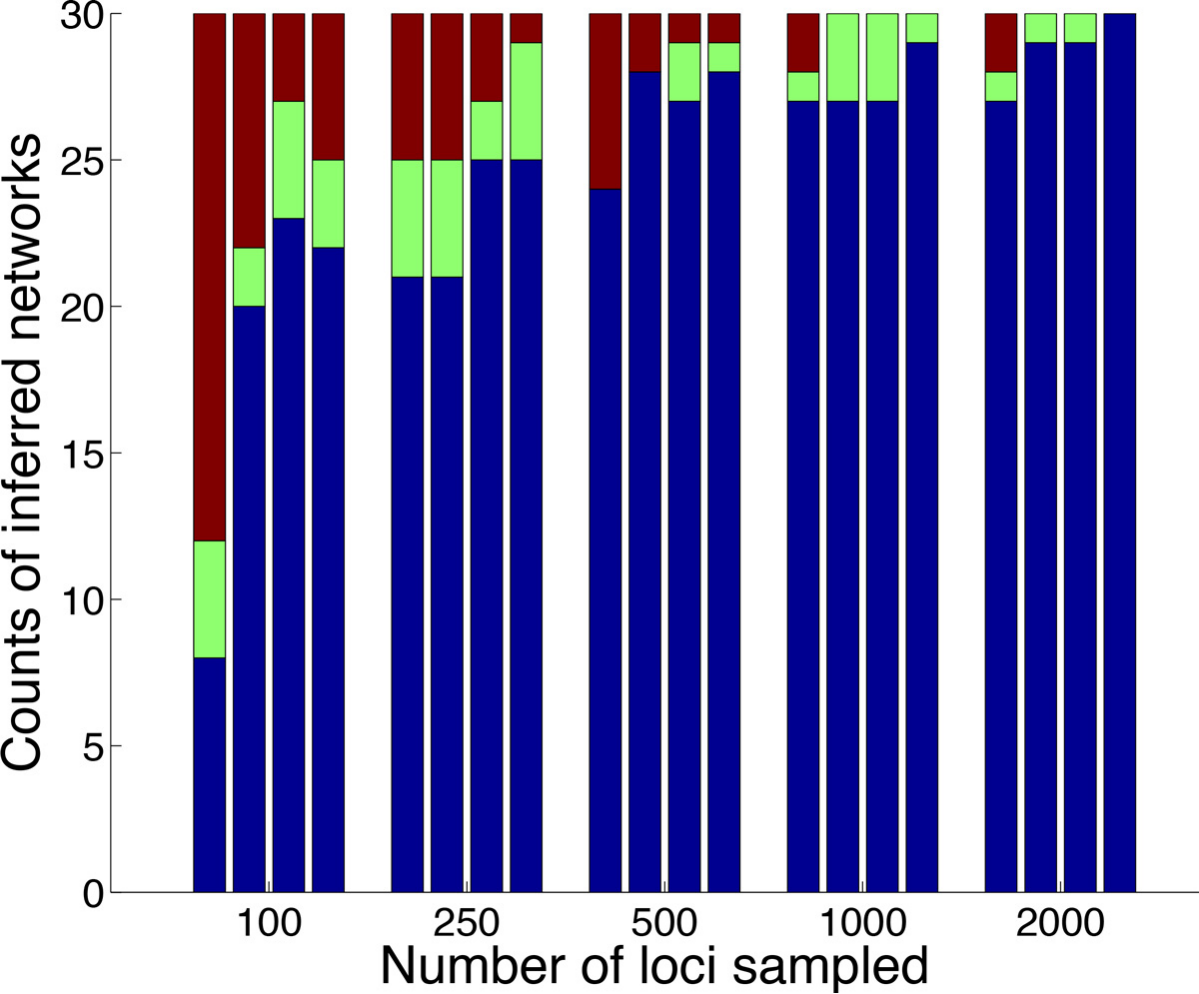
**Accuracy of the method on simulated data**. For every number of loci, the rightmost bar corresponds to inference from true gene trees and the other three bars, from left to right, correspond to inference from estimated gene trees from sequences of lengths 250, 500 and 1000, respectively. The dark blue region corresponds to the number of times the true network was returned as the optimal network after the search. The green region corresponds to the number of times the true network is not the optimal network found by the search, but is the optimal one among the top 5 species networks after optimizing their branch lengths and inheritance probabilities under maximum pseudo-likelihood. All other scenarios are represented by the maroon region.

We ran our method on both the true gene trees and estimated gene trees to infer species networks. The number of reticulations was set to the true value 2. For each dataset, the search was performed 5 times starting from the optimal species tree under the MDC criterion [41]. During the search, the top 5 species networks with highest pseudo-likelihood were saved. After that, we optimized the branch lengths and inheritance probabilities of those top species networks under maximum pseudolikelihood (see the discussion above for the rationale of doing this step). Note that the true network is uniquely encoded by its triples in this case. The results are shown in Figure 4.

Overall, except for the hardest cases (very short sequences and a small number of gene trees), the method made very accurate inferences. As expected, the accuracy improves when the number of loci increases. When true gene trees are used, even data sets with the smallest number of loci yield good results. When estimated gene trees are used, as expected, overall, the inferred species networks from gene trees estimated from longer sequences are more accurate. For the smallest number of loci (100), using gene trees estimated from the shortest sequences (of length 250) results in performance that is much worse than using those estimated from longer sequences (of lengths 500 and 1000). However, the improvement in the accuracy of the inferred species networks gained by using gene trees estimated from longer sequences gets smaller when the number of loci increases. When comparing the results based on true gene trees to those based on estimated gene trees, we observe that using true gene trees is only significantly better when the number of loci is small and the gene trees are estimated from short sequences. In particular, for sequence lengths 500 or 1000 and 1000 gene trees, which are realistic sizes of phylogenomic data sets, the method has 100% accuracy (when coupled with the optimization post-processing step) under our simulation settings.

Finally, we investigated the running time of the method. Given that the time of the search is affected by various factors, we focused here on the running time of computing the pseudo-likelihood of networks of varying sizes. We first used PhyloGen [42] to generate random species trees with 20, 50, 100, 150 and 200 taxa. Then, for each species tree, we randomly added 1, 2, 3, 4 and 5 reticulations (it is important to note that currently computing the full likelihood of networks of these sizes is infeasible). More specifically, to add a reticulation to a species network, we selected two edges uniformly at random and added an edge between their midpoints from the higher one (closer to the root) to the lower one (farther from the root) to avoid creating a cycle. The lower one became a new reticulation node to whose incoming edges inheritance probabilities were assigned uniformly at random. Then, the program ms [38] was used to generate one gene tree within the branches of each species network.

**Figure 5:**
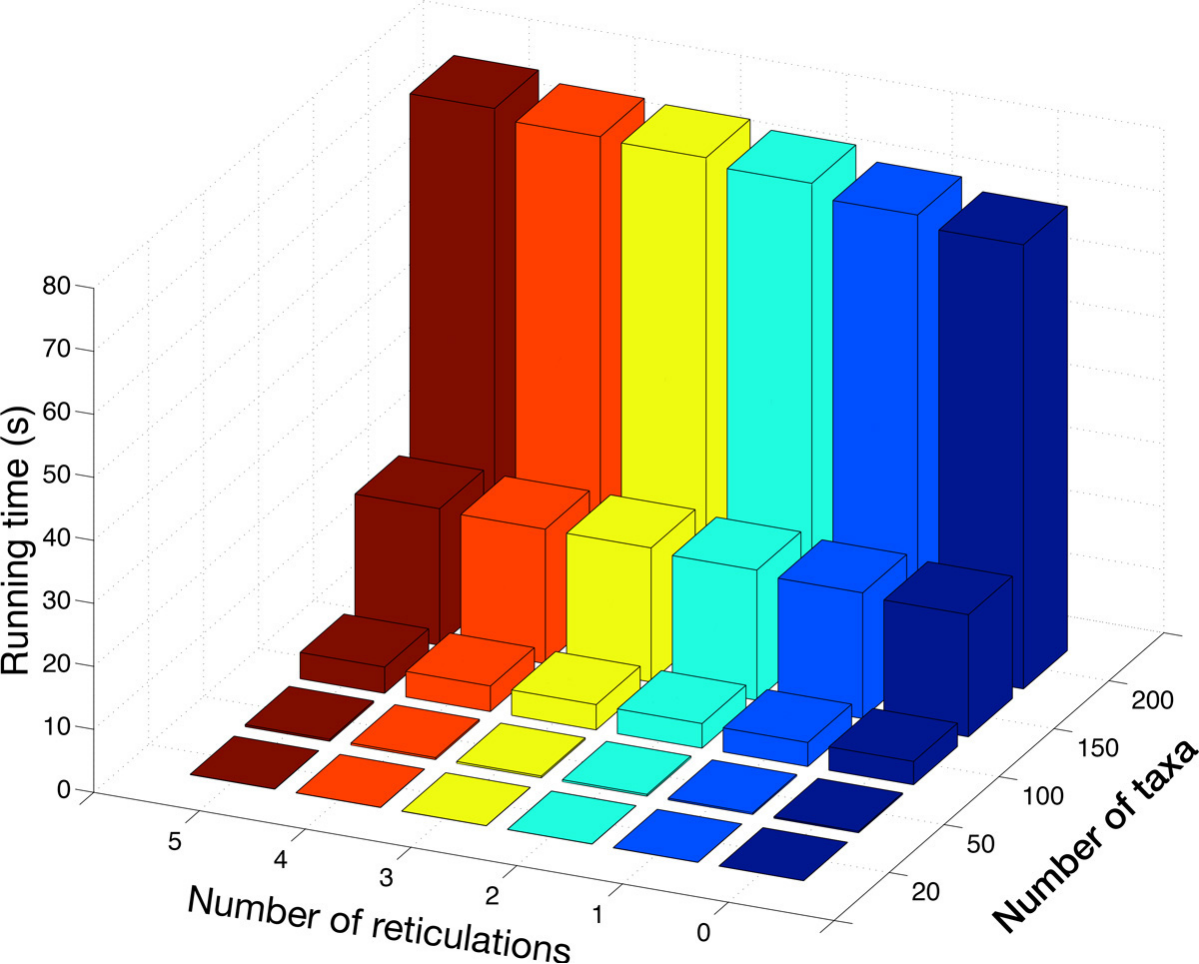
**Running time of computing pseudo-likelihood of a species network**. We varied the species networks by the number of taxa and the number of reticulations. The running times are reported in seconds.

We ran our method in parallel on 8 cores on a system with a 2.83-GHz processor. The results are shown in Figure 5. Overall, computing the pseudo-likelihood of a species network is very fast. It only took around 0.02 seconds for species networks with 20 taxa and 0.25 seconds for species networks with 50 taxa. It is not surprising to see that the running time is dominated by the number of taxa *n*, since that directly determines the number of triples. Further, the running time of computing the probability of a triple increases with the number of taxa of the species network in general. On the other hand, we can see from the figure that for a fixed number of taxa the running time increases ever so slightly with the number of reticulations in the species networks. It is very different from computing the full likelihood, where the number of reticulations and the configurations of the reticulations significantly affect the running time of the likelihood computation [21].

## Discussion

In a recent study, Fontaine *et al*. reported on hybridization and extensive introgression in the *Anopheles gambiae *complex [14]. Further, they discussed the potential for incomplete lineage sorting to be at play and accounted for it in their analysis. The study highlighted an underlying species tree of the *An. gambiae *complex, along with added reticulation edges to capture hybridization. However, Clark and Messer argued that "given that the bulk of the genome has a network of relationships that is different from this true species tree, perhaps we should dispense with the tree and acknowledge that these genomes are best described by a network" [43]. This is just one of the most recent studies in an increasingly large body of work that calls for (i) accounting for ILS when hybridization detection is conducted, and (ii) using networks, rather than trees, to model evolutionary relationships. Indeed, networks encompass trees and provide a more expressive model for reticulate evolutionary histories [44].

Along with coworkers, we recently introduced the first maximum likelihood method for inferring general phylogenetic networks while accounting for ILS [24]. While the method produces very good results in terms of the evolutionary relationships it infers, its computational requirements, particularly those of computing the likelihood of a phylogenetic network candidate, remain a major bottleneck that limits its applicability to very small data sets. In this work, we introduced a pseudo-likelihood model of phylogenetic networks that is based on the rooted triples they induce and inspired by the work of Liu *et al*. on the pseudo-likelihood of species trees [26]. The model, combined with a search heuristic, yields a method for phylogenetic inference that is computationally orders of magnitude more efficient than inference under full likelihood and that produces very good inferences.

**Figure 6:**
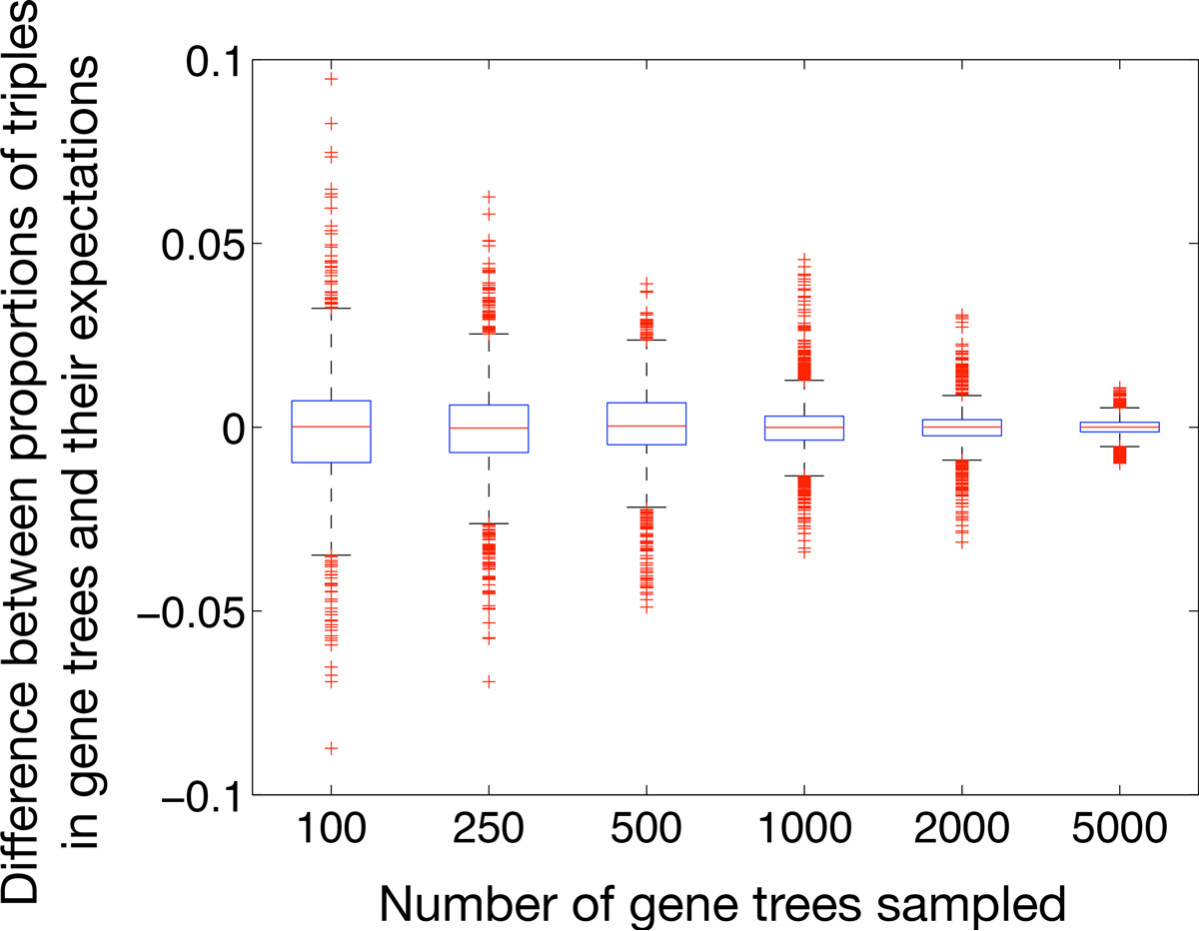
**Convergence of the proportions of rooted triples in gene trees to their expectations**. Every point is the empirical frequency of a triple minus the (theoretical) expectation of that frequency.

As stated by Eq. (4), as the number of gene trees goes to infinity, the proportions of rooted triples in gene trees would converge to their expectations. One issue of practical implications concerns the rate at which this convergence occurs in practice. To explore this issue, we used true gene trees generated in our simulation study, and for every number of loci (100, 250, 500, 1000 and 2000), we randomly selected one dataset out of 30. Then within the branches of the same model species network, we simulated one more set of gene trees of size 5000. For each set of gene trees, we computed the proportions of all rooted triples in gene trees and their expectations and plotted their differences. The results are shown in Figure 6. Clearly, the results show good convergence and helps explain the good performance in the simulation results above. It is important to note that obtaining thousands of loci in phylogenomic analyses is becoming very feasible, particularly that for the purposes of these analyses, a locus can be taken to be any non-recombining genomic region. That is, gene trees in these analyses do not have to be estimated from protein-coding genes, but rather from recombination-free genomic regions regardless of their "coding" status.

An advantage of this method in terms of efficiency is that the running time of the inference depends in a minor way on the number of gene trees. More specifically, after the gene trees *G *are read, *ρ*(*t, G*) is computed only once for all possible rooted triples *t *and the results are saved. Then during the search afterwards, *ρ*(*t, G*) are only constants when computing pseudo-likelihood of species networks.

The major drawback of the method is that not all phylogenetic networks are uniquely encoded by their systems of rooted triples. That is, some systems of rooted triples can encode more than a single network. In these cases, the convergence result given above does not guarantee that the true network is identified; rather, it implies that a network that is equivalent to the true one under rooted triples (potentially the true network itself) might be identified in the search. When such a scenario arises, using different types of data or an alternative criterion to evaluate the identified networks might help to identify the true network.

## Conclusions

Inference of phylogenetic networks based on pseudo-likelihood is very fast and produces very accurate results, thus providing an approach that scales up evolutionary history inference in the presence of hybridization and incomplete lineage sorting to much larger data sets than is currently feasible. Under certain conditions, the true reticulate evolutionary history might not be identifiable from the set of rooted triples. Research into identifiability issues with respect to phylogenetic networks is beginning to emerge [32,45,46], but much more work is needed in this area, particularly for the phylogenetic network model employed here (which accounts for ILS) and data other than gene tree topologies.

## Abbreviations

ILS: incomplete lineage sorting

GTR: general time-reversible

## Competing interests

The authors declare that they have no competing interests.

## Authors' contributions

YY and LN conceived of the study, designed the methods, analyzed the data, and wrote the manuscript. YY implemented the methods and ran the analyses.
